# Bee Products and Colorectal Cancer—Active Components and Mechanism of Action

**DOI:** 10.3390/nu15071614

**Published:** 2023-03-27

**Authors:** Justyna Moskwa, Sylwia Katarzyna Naliwajko, Dominika Dobiecka, Katarzyna Socha

**Affiliations:** Department of Bromatology, Faculty of Pharmacy with the Division of Laboratory Medicine, Medical University of Białystok, 15-222 Białystok, Poland

**Keywords:** propolis, bee honey, bee pollen, royal jelly, bee venom, colon cancer

## Abstract

Colorectal cancer is one of the most common malignancies in the world. Lifestyle and eating patterns may have a significant impact on the prevention of this type of cancer. Bioactive food ingredients influence the gut microbiome and can have a protective effect. Bee products (honey, propolis, royal jelly, and bee venom) or pharmacologically active fractions obtained from them are widely used in many fields of medicine, pharmacy, and cosmetics. Some evidence suggests that bee products may have anti-cancer potential. The main bioactive components with anti-colon cancer potential from propolis and bee honey are polyphenols such as pinocembrin, galangin, luteolin, CAPE, Artepilin C, chrysin, caffeic, and p-coumaric acids. This review is focused on the new data on epidemiology, risk factors for colon cancer, and current reports on the potential role of bee products in the chemoprevention of this type of cancer.

## 1. Introduction

The search for new natural methods for enhancing the body’s immunity and supporting anti-cancer therapy, with less invasive potential and significant effectiveness, is a dominant research trend. Products with such properties include bee products such as honey, propolis, beebread, royal jelly, pollen, and bee venom. Bee products have been used worldwide as traditional medicines for thousands of years to treat various forms of diseases [1,2]. A number of studies confirmed that bee products have many active ingredients in their chemical composition and have shown an extensive spectrum of biological activities such as antibacterial, antiviral, anti-inflammatory, antioxidant, anti-mutagenic, and anticancer [3,4,5,6,7,8]. Apitherapy consists in treating with products made of various combinations of honey, propolis, royal jelly, bee pollen, beebread, and bee venom. After separation and biological standardization, these products are the active ingredients of many medicines [9,10]. Bee products or pharmacologically active fractions obtained from them are widely used in many fields of medicine and pharmacy as pharmacopoeia raw materials, dietary agents, or cosmetics [11].

Treating and preventing cancer is a global challenge. According to the World Health Organization (WHO), one of the most common cancers in 2020 was colorectal cancer (CRC)—10%. The diet can have a significant impact on health; studies report that changes in dietary habits can help prevent cancer in 30–50% [12,13]. Oncology patients often use complementary and alternative medicine (CAM) in addition to conventional treatment. Recent systematic review results showed that an average of 51% of cancer patients used CAM [14]. Bee products have attracted much interest in cancer prevention due to their high content of biologically active substances, lack of toxicity or side effects [15]. Münstedt et al. reported that many books recommend apitherapy for cancer prevention, but no studies provide specific data in comparison to what is known from clinical studies on bee products [7]. Therefore, in the present review, we discuss the new data on epidemiology, risk factors of CRC, and current reports on the role of bee products in the chemoprevention of this type of cancer.

Considering the importance of natural bee products, this review aims to update the current state of knowledge on the anticancer activity of bee products, i.e., propolis, honey, bee pollen, royal jelly, beebread, and bee venom in relation to colon cancer as one of the most common diet-related cancers.

**Figure 1:**
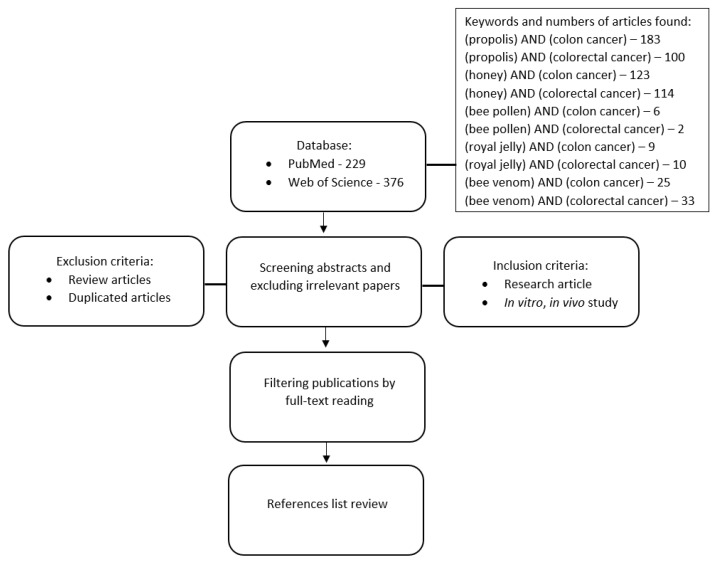
Flowchart showing methodology approach.

A literature search was conducted up until November 2022, using databases including PubMed and Web of Science. The search terms were addressed using the following keywords: “propolis and colon cancer”, “honey and colon cancer”, “bee pollen and colon cancer”, “bee pollen and cancer, “royal jelly and colon cancer”, “bee venom and colon cancer”, “propolis and colorectal cancer”, “honey and colorectal cancer”, “bee pollen and colorectal cancer”, “bee pollen and colorectal cancer, “royal jelly and colorectal cancer”, “bee venom and colorectal cancer” (Figure 1).

## 2. Bee Products—Anti-Colon Cancer Potential

### 2.1. Colon Cancer—Epidemiology and Risk Factors

Colorectal cancer (CRC), also known as colon cancer, bowel cancer, or rectal cancer, is the development of cancer from the colon or rectum (parts of the large intestine). According to the World Health Organization GLOBOCAN database, CRC is the third most common cancer in men and the second most common in women worldwide. The overall CRC incidence was 1,931,590 and mortality was 935,173 worldwide [16]. Data show that the overall survival at 5 years after diagnosis is approximately 60%, considering all stages of the disease [17]. About 1,823,278 of new cases of CRC are estimated to be diagnosed in 2040, while the highest increase is estimated in Africa (96.0%) and Asia (75.5%) and the lowest is in Europe (27.1%) [18].

CRC’s incidence is approximately 25% higher in males than in females and is approximately 20% higher in African Americans than in Whites [19]. Most cases of CRC are mainly dependent on lifestyle factors, and only a small percentage of cases are dependent on genetic factors (Lynch syndrome, Crohn disease). Major risk factors include obesity, diabetes mellitus and insulin resistance, red and processed meat consumption, high alcohol consumption, smoking, and inflammatory intestinal conditions [20].

CRC usually begins with a noncancerous proliferation of epithelial cells lining the colon or rectum of the gastrointestinal tract, resulting in a polyp most often as a result of mutations in the Wnt signaling pathway that increase signaling activity. These mutations can be inherited or acquired and most likely occur in the stem cells of the intestinal crypts. Polyps are common, detected in about half (including serrated polyps) of average-risk individuals 50 years of age or older undergoing colonoscopy. However, fewer than 10% of polyps are estimated to progress to invasive cancer [21,22], a process that usually occurs slowly over 10 to 20 years and is more likely as polyps increase in size. The most common form is adenocarcinoma, which accounts for 90–95% of all human colorectal malignancies [23].

Obesity increases the prevalence of CRC, even among those who are physically active [24]. The data suggest that obesity is associated with a 50% higher risk of colon cancer and a 25% higher risk of rectal cancer in men, whereas obese women have about a 10% increased risk of colon cancer and no increased risk of rectal cancer [25]. Mechanisms explaining the association between obesity and CRC include: inflammation, insulin resistance, and the release of growth hormones by adipose tissue [26]. Studies have shown that weight loss after bariatric surgery was associated with a 39–60% reduction in the risk of CRC mortality [27,28,29].

Type 2 diabetes mellitus is associated with an increased risk of CRC. A number of meta-analyses indicated that the risk of colon cancer among diabetics was approximately 38% higher than nondiabetics (RR 1.38, 95% CI 1.26–1.51), and for rectal cancer, it was 20% higher (RR 1.20, 95% CI 1.09–1.31) [30]. A study by Kanadiya et al. [31] showed that among 405 patients with diabetes and 3038 subjects without diabetes (who underwent their first colonoscopy), the risk associated with a higher incidence of colorectal adenoma (OR = 1.35) was higher in diabetics (29.3%) compared with non-diabetics (23.9%). The association between type 2 diabetes and CRC may be related to an increase in IGF-1 factor (by hyperinsulinemia), which is responsible for the intensification of epithelial cell dysplasia and induces CRC proliferation [32]. Other mechanisms include the intake of metformin, which is responsible for the activation of AMP-activated protein kinase (AMPK). Research suggests that AMPK is activated by a low dose of metformin and inhibits the formation of irregular crypt foci (ACFs), which are a specific marker of CRC [33,34].

Consumption of red and/or processed meat is associated with an increased risk of CRC [35]. A recent study for the World Cancer Research Fund estimated that for every 50 g of processed meat consumed per day, the risk of CRC increases by approximately 16%, and for every 100 g of red meat consumed per day, it increases by approximately 12%. In 2015, the International Agency for Research on Cancer classified processed meat as “carcinogenic to humans” and red meat as “probably carcinogenic to humans,” largely based on the evidence related to CRC risk [36]. Research results highlight the role of heme iron in the promotion of colon cancer by red meat and suggest that heme iron could initiate carcinogenesis through lipid peroxidation.

A number of studies confirm a connection between high alcohol consumption and an increased risk of CRC. Durko et al. [37] showed that an intake of 30 g/day of alcohol is associated with a 16% increase in CRC risk, whereas an intake of 45 g/day elevates this risk by 41%. A recent meta-analysis indicated that alcohol consumption (up to two drinks per day) was associated with a slightly lower (8%) risk than no consumption/occasional consumption, whereas very heavy drinking (more than three drinks per day) was associated with a 25% higher risk [38].

The meta-analysis reported that smoking increases the risk of CRC in a dose-dependent manner with the duration and intensity of smoking and provides evidence that quitting smoking reduces CRC risk. The study showed that mechanisms are associated with the MSI pathway, characterized by MSI, CIMP, and BRAF mutations. The risk of CRC is increased by 25–30% in smokers of 40 cigarettes per day or in those who smoke for 50–60 years [39].

Research highlighted factors associated with a decrease in the incidence of CRC; these include regular physical activity; a diet high in fruits, vegetables, and fish; a high intake of fiber; and increased intake of dietary or supplemental calcium, magnesium, and products with a high content of antioxidants [40]. A diet rich in bioactive compounds may play an important role in the prevention of CRC. Dietary intervention demonstrated by Citronberg et al. (2013) showed that daily supplementation for 28 days with 2.0 g ginger in 20 patients at a high risk of colorectal cancer resulted in a decreased proliferation rate and increase in apoptosis and differentiation relative to proliferation in the differentiation colonic zone of the crypts [41]. The in vitro study suggests that combination ginger extract with Gelam honey inhibits cell proliferation and caused DNA damage, cell cycle arrest, and induction of apoptosis in colon cancer cells HT29 [42].

### 2.2. Propolis

Propolis is a waxy, resinous substance produced by bees from resin collected from trees and shrubs, which they then combine with beeswax and secretions from salivary glands rich in many enzymes. Raw propolis is not suitable for human consumption because it is very viscous and poorly soluble in water, but extracts contain a wide range of active components, whose concentrations depend primarily on the geographical provenance, season of the year, and the breed of bees. There are some types of propolis: “Poplar” (European, Chinese, North and South American, including Manuka propolis from New Zealand), “Brazilian green” (containing artepillin-C), “Red” (from Cuba, Brazil, Mexico), “Birch” (from Russia), “Mediterranean” (Greece, Crete, Sicily, Malta), “Pacific” (from Okinawa, Taiwan, Indonesia), and “Clusia” (from Cuba and Venezuela) [43]. The main components of propolis are fatty, aliphatic (24–26%) and aromatic acids (5–10%), flavonoids (18–20%), alcohols and terpenes (2–3.3%), sugars (15–18%), esters (2–6%), vitamins (2–4%), and microelements (0.5–2%) [44]. In the composition of propolis, about 300 compounds have been identified from which the most active compounds include chrysine, caffeic acid phenethyl ester (CAPE), quercetin, p-coumaric acid, kaempferol, pinocembrin, galagin, artepillin C [45,46]. The health-promoting effects of polyphenols are still debated and require good quality research. A study by Curti et al. on the bioavailability of brown propolis in acute and long-term oral administration conditions of C57BL/6 wild-type mice showed that there is rapid absorption and metabolization of galangin, followed by adaptation of the first-line antioxidant defense system (SOD-1 increased significantly) [47]. Studies by other authors have shown that Pinocembrin and pinostrobin, as one of the main flavonoids found in propolis, have low bioavailability after oral ingestion in rats. The bioavailability of S-pinocembrin and R-pinocembrin was 43.2% and 57.8%, while that of S-pinostrobin and R-pinostrobin was only 1.83% and 13.8%, after oral administration in rats [48].

Propolis has many health benefits, including antioxidant, anti-inflammation, anti-viral antimicrobial, antifungal, anticancer, antidiabetic, anti-Alzheimer’s, and liver protection [49]. Currently, the standardization of propolis is a challenge, however, its biological activity makes it a product with a wide potential especially in anticancer therapy.

#### Anti-Colon Cancer Potential of Bee Propolis

A number of in vitro and in vivo studies confirm the anticancer activity of propolis or its active components against colon cancer cell lines (Table 1). The extracts of Polish, Brazilian Red, New Zealand, and Serbian propolis have shown high levels of cytotoxicity on HCT-116 colon cell lines [50,51,52,53,54,55,56,57]. Other studies demonstrated that Brazilian and Chinese propolis extracts inhibited the growth of human colon carcinoma HCT116, HT29, and SW480 cell lines, with IC50 values in the range of 4–41 μg/mL. Additionally, Chinese propolis extract caused a dose-dependent increase in the cellular mRNA levels of p21CIP1 and p53 in the HCT116 cell line [57]. The study by Russo et al. showed that the Chilean propolis protects normal cells from oxidative and also reduces the vitality of Caco-2 colon adenocarcinoma cells through the induction of DNA damage [58]. The anticancer properties of the Portuguese propolis in different fractions (hexane, chloroform, and ethanol) on the human colon carcinoma cell line HCT-15 were investigated by Valença et al. [59]. The study indicated that all propolis samples caused a cytotoxic effect against HCT-15 cells, in a dose- and time-dependent way. Chloroform fraction was found to be the most active, both in terms of the inhibition of viability and cell death. Data also show that this cytotoxicity involves a disturbance in tumor cell glycolytic metabolism by a decrease in glucose consumption and lactate production [59]. Azarshinfam et al. showed that Iran propolis extract increases Bax pro-apoptotic gene expression, decreases Bcl-2 anti-apoptotic gene expression, and induces apoptosis in Ht-29 cancer cells. Moreover, propolis extract combined with layered double hydroxide (LDH) nanoparticles (NPs) significantly enhances its efficacy (in all cases, *p* < 0.05) [60].

Propolis induces an antitumor response alone or in conjunction with other drugs. Frión-Herrera et al. [61] confirmed that brown Cuban propolis (CP) and its main component, nemorosone, increase the cytotoxic effect of doxorubicin (Dox) in the human colorectal adenocarcinoma cell line (WT) and particularly in its resistant variant (LoVo-Dox), activating apoptosis mechanisms by cell cycle arrest in G0/G1 phase. Moreover, CP-Dox treatment in LoVo cell lines was preceded by increased ROS levels and the alteration of mitochondrial membrane potential. A recent study showed the protective effect of propolis against potassium bromate toxicity by decreasing lipid peroxidation and reversing the main molecule levels (caspase-3, caspase-8, caspase-9, cytochrome-c, TRAIL, and APAF) in the intrinsic and extrinsic pathway of apoptosis in CCD 841 normal colon cells [62]. Furthermore, the reduction of allergenic molecules in propolis via biotransformation did not change the antioxidant and protective effects of propolis, and it is suggested as a potential therapeutic molecule in the prevention of colon cancer [62]. An interesting study by Cho et al. [63] ascertained the ability of a propolis supplement to modulate intestinal neoplastic development in C57BL/6J-ApcMin/+/J mice in the lean and obese state. The study showed a statistically significant decrease in the number of adenomas in mice fed a control diet with the propolis supplement (61.8 ± 10.6 vs. 35.3 ± 7.6, *p* = 0.008); moreover, mice on a propolis-supplemented Western diet did not gain excessive body weight. Despite the fact that propolis shows many biological properties, there is a problem with its standardization and limited oral bioavailability research results in this area have been carried out [64]. The results demonstrated a considerable enhancement in standardized propolis solubility, with a controlled release profile in different gastrointestinal tract environments and increased anticancer activity of the newly developed propolis-loaded nano-in-microparticles (NIMs) against human liver cancer (HepG2) and human colorectal cancer (HCT 116) cells. The recent in vivo study examined by Sameni et al. [65] showed that the administration of propolis extract alone significantly reduced the number of aberrant crypt foci (ACFs) compared to the AOM group (cancer control group), which indicated an inhibitory role in the onset or progression of CRC, but its therapeutic effect was lower than 5FU in this study. However, various doses of propolis combined with 5FU significantly reduced the number of ACFs compared to the treatment with 5FU alone, which indicated its synergistic effect with 5FU in mouse model Balb/c. The protective influence of propolis on the process of colon carcinogenesis in animal models was also observed by other authors [66,67,68,69,70]. A number of in vitro and in vivo studies confirmed the efficacy of the anti-cancer effects of propolis and encourage researchers to conduct clinical trials. One of the latest planned studies is a clinical trial (Iranian Registry of Clinical Trials IRCT20190708044154N1) to evaluate the efficacy of propolis supplementation (900 mg/day for 6 weeks) in patients with irritable bowel syndrome (IBS), which is one of the risk factors of CRC [71]. The study showed a significant reduction in the overall score of IBS symptoms, the severity of abdominal pain, and the frequency of abdominal pain with propolis administration as compared to placebo (*p*-value < 0.05). The authors reported that propolis may be used as adjunctive therapy in IBS to reduce abdominal pain. On the other hand, the pilot study by Ishikawa et al. has not confirmed the effectiveness of propolis studies in preventing changes occurring during the early stages of colon cancer. The results showed that treatment with Brazilian propolis extract (300 mg/day for 3 months) significantly increased the mRNA level of cyclin D1 in the sigmoid colon mucosa, increased the myocardial band from creatine phosphokinase (CPK) activity, and may have adverse effects on muscle tissue [72].

**Table 1:** The main active components from propolis with anti-colon cancer potential.

Compounds	Type of Cancer	Type of Study	Activity	References
Pinocembrin galagin luteolin	Colon cancer	In vitro/HTC-116	↑ cytotoxic activity	
			↑ apoptosis	Vukovic et al. 2018 [73]
			↓ superoxide anion radical ↓ nitrites	
CAPE	Colon cancer/ Gastric adenocarcinoma	In vitro/HTC-116, HT-29, AGS, SW480, CT26 In vivo/male Wistar rats	↑ cytotoxic activity ↑ genotoxic activity ↑ caspase-3/7 ↓ ROS ↑ G1 phase ↓ cyclin D1, c-myc ↓ beta-catenin/T-cell factor ↓ formation of (ACF) and tumors	Gajek et al., 2020 Xiang et al., 2006 Fraser et al., 2016 Liao et al., 2003 Borrelli et. al., 2002 [74,75,76,77,78]
CAPE-pNO2	Colon cancer	In vitro/HT-29 In vivo/Male BALB/c nude mice	↑ p53 ↑ caspase-3 ↑ Bax ↑ P38 ↑ CytoC ↑ P21Cip1 ↑ P27Kip1 ↓ CDK2, c-Myc ↑ G0/G1 phase ↑ inhibition of tumor growth ↓ VEGF	Tang et al., 2017 [79]
Galangin	Colon cancer	In vitro/HCT-15, HT-29	↑ cytotoxic activity ↑ caspase 3, 9 ↑ DNA condensation ↓ mitochondrial membrane potential	Ha et al., 2013 [80]
Artepilin C Baccharin Drupanin	Colon cancer	In vitro/DLD-1	↑ TRAIL, FasL ↑ miR-143 ↓ MAPK/Erk5 ↓ c-Myc	Kumazaki et al., 2014 [81]
Artepilin C	Colon cancer, Liver hepatoblastoma	In vitro/ Caco-2 HepG2	↑ G0/G1 phase ↓ cyclin D/cyclin-dependent kinase 4 ↑ Cip1/p21, Kip1/p27	Shimizu et al., 2005 [82]
Mucronulatol	Colon carcinoma	In vitro/HCT8	↑ sub-G1 phase ↑ Cip1/p21, Kip1/p27 ↓ cyclin E, CDK4	Diaz-Carballo et al., 2008 [83]
Plukenetione A	Colon cancer wild-type, -FU-resistan, SN38-resistant Oleocecal carcinoma wild-type, SN38-resistant, Raltitrexed-resistant	In vitro/HT29 WT, HT29 24R, HT29 SN3, HCT8 WT, HCT8 SN38, HCT8 ICID	↑ G0/G1 phase ↑ DNA fragmentation ↓ expression of topoisomerase II-beta, ↓ EGF receptor	Diaz-Carballo et al., 2008 [84]
Chrysin	Colon cancer	In vitro/ SW48, SW480, SW620, HT-29, HCT-116	↓ viability ↑ LC3-II autophagy marker ↑ ROS ↓ protein kinase B(Akt) ↓ rapamycin (mTOR)	Lin et al., 2018 [85]

↑—increase; ↓—decrease; CAPE—caffeic acid phenethyl ester; ROS—reactive oxygen species; ACF—aberrant crypt foci.

### 2.3. Bee Honey

Bee honey is the natural sweet product produced by Apis mellifera honeybees by combining with their own specific substances (plant nectar or the secretions of living parts of plants or the excretions of insects sucking the sap of living parts of plants), stored, evaporated, and left to ripen in the combs. For centuries, honey has been used in folk medicine for its medical and health promotion properties. The chemical composition of honey depends on the type and species of plants from which the bees collect nectar or honeydew. More than 300 active constituents have been discovered in different types and varieties of honey [3]. The main ingredients of bee honey are sugars (about 76%), water (<20%), nitrogen compounds, organic acids, essential oils, pigments, vitamins, and other active substances such as flavonoids, phenolic acids, and carotenoids [86]. Flavonoids and phenolic acid are among the most active compounds of honey that contribute to its antioxidant and anticancer properties. According to different studies, the concentration of flavonoids in honey is about 20 mg/kg, and it differs depending on the botanical origin of the honey [85]. The most common flavonoids and phenolic acids determined in honey include quercetin, kaempferol, myricetin, chrysin, luteolin, apigenin, diosmetin, pinocembrin, hesperetin, naringenin, epicatechin, catechin, epigallocatechin, benzoic acid, vanillic acid, syringic acid, salicylic acid, gallic acid, ellagic acid, affeic acid, p-coumaric acid, ferulic acid, and sinapic acids, and others depending on the botanical origin [3]. Studies by Karim et al. [86] on the bioavailability of honey showed that supplementation with buckwheat honey containing approximately 1.171 mg/g of polyphenols resulted in a significant increase in plasma antioxidant and reduced capacities and concentration 2 h after ingestion, and it remained at a high level for 6 h. Another study evaluating the bioavailability of Manuka honey polyphenols and their antioxidant and antitumor capacity in in vitro gastrointestinal digestion in human HCT-116 colon cancer cells showed that total polyphenols, total flavonoids, and TAC were significantly (*p* < 0.05) reduced after in vitro digestion. Moreover, both Manuka honey before and after digestion showed similar effects in inducing intracellular ROS production and inhibiting the ability to form colonic [87]. Honey is a well-known medicinal agent due to its many biological and pharmacological properties. It was observed to have antioxidant, antimicrobial, anti-inflammatory, wound healing, anti-mutagenic, and anti-tumor activities [88,89,90,91,92]. There are many reports in the literature confirming the antibacterial and anti-inflammatory effects of honey. Studies showed that honey can reduce the presence of infection-causing bacteria in the gut, such as Salmonella, Escherichia coli, and Clostridium difficile [93,94], and it has been reported that honey causes increased growth of probiotic Bifidobacterium and Lactobacillus species [95,96,97]. Other studies indicate that honey may have immunomodulatory effects through anti-inflammatory effects by downregulating inflammatory transcription factors and cytokines and leads to a reduction in the severity of chronic inflammatory diseases [98,99]. As the gut microbiota may play a role in the development of the tumorigenesis process, honey showing probiotic and anti-inflammatory properties appears to be a good opportunity for prevention and treatment; however, further clinical studies are required.

#### Anti-Colon Cancer Activity of Bee Honey

Recently, there has been extensive research on the use of natural and synthetic drugs in the prevention of CRC. In this regard, bee honey may be an important alternative due to its high content of many active substances showing chemotherapeutic activity (Table 2). Cianciosi et al. [87] studied the influence of in vitro gastrointestinal digestion on the anticancer activity of Manuka honey (MH). The results showed that the total phenolic and flavonoids content in digested MH significantly decreased compared to undigested MH, but, interestingly, the antiproliferative effects against HCT-116 after the treatment of digested and undigested MH were similar regarding intracellular ROS production and the inhibition of colony formation. Afrin et al.’s [100] study showed that MH (0–20 mg mL^−1^) induced a strong reduction in viability of HCT-116 colon cancer cells by decreasing the proliferation ability, cell cycle arrest as well as apoptosis, and cell cycle regulatory gene and protein expression. However, it did not cause a toxic effect in the normal LoVo cell line. In addition, the induction of apoptosis was demonstrated by increasing expression of p53, cleaved-PARP, and caspase-3. MH induced cell cycle arrest in the S phase in HCT-116 cells, and simultaneously, in LoVo cells, it occurred in the G2/M phase through the modulation of cell cycle regulator genes (cyclin D1, cyclin E, CDK2, CDK4, p21, p27, and Rb). The expression of p-Akt was suppressed while the expression of p-p38MAPK, p-Erk1/2, and endoplasmic stress markers (ATF6 and XBP1) was increased for apoptosis induction. Furthermore, the study demonstrated that MH induces HCT-116 and LoVo cell death by enhancing oxidative stress, as well as by regulating the energy metabolism aerobic and anaerobic pathways and inhibiting the metastatic capacity (MMP-2 and MMP-9) [101]. Interestingly, the other studies by these authors showed that MH synergistically enhanced the chemotherapeutic effects of 5-fluorouracil (5-FU) by reducing cell proliferation (HCT-116) through the suppression of EGFR, HER2, p-Akt, and p-mTOR expression and promoting apoptosis by the modulation pro-apoptotic (p53, Bax, Cyto c, FasL caspase-3, 8, 9, and cleave-PARP) and anti-apoptotic (Bcl-2) markers. Additionally, it was shown that MH, synergistically with 5-FU, caused cell cycle arrest and influenced the anti-metastasis effects of 5-FU by decreasing the migration ability and suppressing the expression of MMP-2 and MMP-9 [102].

The synergistic effect of 5-FU and Gelam honey (GH) was also confirmed by a number of other studies. The research showed that GH enhanced the cytotoxic and apoptotic effects of 5-FU on the colon cancer HT-29 cell line [103,104]. Other studies of GH combination with ginger demonstrated inhibited growth in most HT-29 cells, while GH alone decreased the viability dose-dependently (IC50 88 mg/mL). Additionality, GH with ginger treatment decreased the gene expressions of Akt, mTOR, Raptor, Rictor, β-catenin, Gsk3β, Tcf4, and cyclin D1 while the cytochrome C and caspase 3 genes were shown to be upregulated [105]. A similar effect has also been shown by Tahir et al. [42]. GH combined with ginger treatment on HT-29 inhibited the growth of HT29 colon cancer cells by inducing early apoptosis, modulating the expression of genes involved in the KRAS/ERK/PI3K/AKT pathways, and suppressing inflammation via the NFκB pathway [42].

In vitro and in vivo studies by Das et al. [106] found that multifloral Indian honey in particular showed a significant inhibitory impact on HCT-15 cancer cell growth by restricting cell proliferation, causing apoptosis, and restricting the cell cycle in the G2/M phase. Honey was also shown to alleviate colon cancer in a DMH-induced colorectal carcinogenesis rat model [106].

The in vivo study performed by Jaganathan et al. [107] confirmed that honey with a higher phenolic content and eugenol (one of the phenolic constituents of honey) inhibit the growth of Ehrlich ascites in BALB/c mice. The maximum tumor growth inhibition by honey was found to be 39.98% and 28.88% by eugenol.

Evidence suggests that honey has anti-cancer potential, as confirmed in in vitro and in vivo studies. However, it should be mentioned that its use in cancer prevention may be problematic due to the fact that it contains large amounts of simple sugars, the consumption of which is not recommended in the diet of people with cancer. Although studies by other authors [108,109] have shown that the antiproliferative effect of honey superseded the osmolarity effects of sugars, research in this area should be expanded.

**Table 2:** The main active components from honey with anti-colon cancer potential.

Compounds	Type of Cancer	Type of Study	Activity	References
Eugenol	Ehrlich ascites carcinoma	In vivo/ BALB/c mice In vitro/HTC-15, HT-29	↑ %Tumor growth inhibition ↑ Sub G1 phase ↑ ROS ↑ DNA fragmentation ↓ MMPs ↑ p53 ↑ PARP ↑ caspase 3	Jaganathan, 2010 Jaganathan et al., 2011 [107,110]
5-Hydroxymethyl-2-furfural	Colon cancer Breast cancer	In vitro/ HT29, MDA in silico	Block Aquaporin-1 ↓ migration	Chow et al., 2020 [111]
Caffeic acid	Colon cancer	In vitro/HCT 15	↓ proliferation ↑ sub G1 phase ↓ colony formation ↑ROS ↓ mitochondrial membrane potential	Jaganathan, 2012 [112]
p-coumaric acid	Colon cancer	In vitro/HCT 15, HT-29	↓ proliferation ↑ sub G1 phase ↓ colony formation ↑ ROS ↓ mitochondrial membrane potential	Jaganathan, 2013 [113]
Polysaccharides isolated from Alhagi honey	---	In vivo/ICR mice treatment cyclophosphamide (chemotherapeutic in colon cancer)	↑ Peyer’s patch count ↑ IL-2, IL-6, TNF-α ↑ SOD ↑ β-defensin ↓ MDA, DAO ↑ p-ERK expression ↓ p-JNK, p-p38	Cai et al., 2021 [114]
3′-Hydroksypterostilben	----	In vivo/ICR mice/azoxymethane (AOM)/dextran sodium sulfate (DSS) model	↓ number of tumors in AOM/DSS-treated mice ↓ nitric oxide synthase ↓ cyclooxygenase-2, ↓ IL-6	Lai et al., 2017 [115]

↑—increase; ↓—decrease; MMPs—matrix metallopeptidases; ROS—reactive oxygen species; SOD—super oxide dismutase; PARP—poly(ADP-ribose) polymerase; MDA—malondialdehyde; DAO—diamine oxidase.

### 2.4. Bee Pollen

Bee pollen is produced by honeybees (Apis mellifera L.) from pollen collected from plant anthers, which is then mixed with nectar or insects’ salivary gland secretions and placed in special baskets on their hind legs (corbiculae). In this form, it goes to the hive, where the honeybees mix it with their saliva and pack it into honeycombs [11,116]. Over 200 compounds have been detected in bee pollen, including proteins (5–60%), essential amino acids, lipids (4–7%), nucleic acids, reducing sugars (13–55%), and crude fiber (0.3–20%). Bee pollen is rich in minerals such as calcium, magnesium, iron, zinc, copper, and vitamins, including 𝛽-carotene, tocopherol, niacin, thiamine, biotin and folic acid, enzymes, and coenzymes. Important compounds are bioactive substances, including saturated and unsaturated fatty acids (1–10%), phospholipids (1.5%), and phytosterols, including 𝛽-sitosterol, β-sitosterol (1.1%), and terpenes. Polyphenols play an important role in the composition of bee pollen, mainly flavonoids (3–8% of dry matter). The most common are catechins, kaempferol, quercetin, and isoramnetin. Bee pollen is also rich in limited carotenoid pigments such as lycopene or zeaxanthin. A number of metabolites contained in bee pollen exhibit antioxidant, anti-inflammatory, anti-carcinogenic, antibacterial, antifungal, hepatoprotective, and anti-atherosclerotic properties, capable of modifying or regulating immune functions [117]. Despite the rich chemical composition of bee pollen, there are few scientific studies on its effect on colon cancer. Wang et al. [118] showed that bee pollen polysaccharides (WRPP) extracted and fractionated from Rosa rugosa inhibited the proliferation of HT-29 and HCT116 cells in a dose-dependent manner in vitro, indicating a potential antitumor activity [118]. An interesting study by Uțoiu et al. [105] showed the enhanced health benefits of pollen by fermentation with a Kombucha consortium. The results demonstrated that the content of bioactive ingredients is higher in fermented pollen and showed a cytotoxic effect (MTT assay) by decreased Caco-2 cells below 10% at concentrations of 20–30 mg/mL [119].

### 2.5. Royal Jelly

Royal jelly is a secretion of the lower pharynx and mandibular salivary glands of honeybees. It occurs in the form of a white-yellowish colloid consisting of about 67% water, 16% carbohydrates, 12.5% protein and amino acids, and 5% fat. The other ingredients include vitamins, minerals, enzymes, and phenols [120]. Approximately 185 organic compounds have been detected in royal jelly. A significant number of them are bioactive compounds, including 10-hydroxy-2-decenoic acid (HDA), adenosine monophosphate oxide (AMP) N1, adenosine, acetylcholine, polyphenols, and hormones, including estradiol, progesterone, prolactin, and testosterone [121]. Royal jelly has been widely used in the pharmaceutical and cosmetic industries as well as functional food. Numerous studies have confirmed its antibacterial activity and antihypertensive, anti-inflammatory, antihypercholesterolemic, and anti-cancer effects in animal models. In addition, clinical trials have shown that it has anti-diabetic properties and has a positive effect on wound healing in diabetic foot ulcers and on benign prostatic hyperplasia [120].

Yang et al. [122] investigated the anti-inflammatory activity of the main component of royal jelly, 10-hydroxy-2-ester (10-HDA) acid, on human WiDr colon cancer cells. The analysis of pro-inflammatory cytokines, receptor antagonist cytokine (IL-1ra), and nuclear factor- kappa B (NF-κB) was analyzed by enzyme-linked immunosorbent assay (ELISA) or Western blotting. The results showed that 10-HDA has an inhibitory effect on the production of pro-inflammatory cytokines IL-1β, IL-8, and TNF-α in WiDr cells. Moreover, it induced the production of IL-1ra in a dose of 0.1–3.0 mM, as a consequence of which IL-1ra limited the production of IL-1β. 10-HDA significantly inhibited the production of IL-8 in a dose-dependent manner at a dose of 0.5–3.0 mM and NF-κB in WiDr cells. The authors indicate that 10-HDA can be used as a chemopreventive agent in carcinogenesis and chronic inflammation; however, further in vitro studies should be performed to determine whether it has anti-inflammatory and anti-cancer properties in the human gastrointestinal tract.

Other interesting studies carried out on Wistar rats confirmed the clinical usefulness of royal jelly as a substance with anti-cancer properties in the prevention and treatment of colorectal cancer. Kaboo et al. [123] tested 60 rats, which were divided into six groups of 10: a control group, two groups receiving royal jelly at a concentration of 300 mg/kg and vitamin E at a dose of 180 mg/kg by gavage once a week, a group receiving 30 mg/kg dimethylhydrazine (DMH) subcutaneously once a week, and two groups treated with DMH, additionally receiving 300 mg/kg royal jelly and 180 mg/kg vitamin E, respectively. Moreover, the cytotoxicity of royal jelly was investigated in the HT-29 cell line. An in vitro study using the MTT test showed that the LC50 of royal jelly was 1.781 mg/mL and the highest level of toxicity was observed at a concentration of 25 mg/mL after 48 h. An in vivo study after a period of 13 weeks showed that rats exposed to DMH with royal jelly simultaneously experienced greater total antioxidant capacity (*p* < 0.05) and less oxidative stress (*p* < 0.05) than rats in the DMH group. Moreover, in the group receiving DMH with royal jelly, a significant decrease in the expression of the nuclear protein of the proliferating cell antigen, the carcinoid antigen, and the platelet-derived growth factor was observed. The overall biochemical indices were significantly better in the group of rats treated with royal jelly, and pathological studies showed less inflammation, congestion, necrosis, and cell proliferation in the colon tissue [124].

### 2.6. Bee Venom

Bee venom, also known as apitoxin, is a complex fluid secreted by the bee venom gland, which is found in the abdominal cavity of bees and is injected into victims using a stinger. It can cause local inflammation and trigger an immune response in the body. Bee venom consists mainly of amphipathic polycationic peptides, including apamines and melittins, enzymes such as phosphatase A2 and low-molecular-weight compounds, e.g., active bioamines. It was used in acupuncture and apitherapy, by injecting the patient with an analgesic, anti-inflammatory agent, as well as for immunotherapy or treatment of Parkinson’s disease. Moreover, bee venom has a number of anti-cancer effects, including radioprotective and antimutagenic properties [120].

In their study, Zheng et al. [124] investigated the antitumor properties of bee venom on the growth of HCT116 and SW480 colorectal cancer cells by activating the death receptors (DR4 and DR5) and suppressing the nuclear factor kappa B. Studies have shown that bee venom significantly inhibited the growth of colon cancer cells by inducing apoptosis and increased the expression of pro-apoptotic proteins such as Bax, caspase-3, caspase-8, and caspase-9 in a dose-dependent manner (0–5 μg/mL). Additionally, treatment with bee venom inhibited the nuclear DNA binding activity of kappa B factor. It has also been proven that combined therapy with bee venom and p50 siRNA transfection or NF-κB PAO inhibitor enhanced the inhibition of cell growth induced by bee venom. Moreover, bee venom inhibited tumor growth in an in vivo study [124].

Another study assessed the cytotoxicity of Apis mellifera venom obtained from A. mellifera syriaca bees and the synergistic effect of its two main biopeptides: melittin (MEL) and phospholipase A2 (PLA2), on colon cancer cell line HCT116. The results showed high cytotoxic activity induced by bee venom and slightly lower cytotoxic activity with MEL or PLA2 alone. The simultaneous administration of both biopeptides significantly increased the cytotoxic effect, which proves the synergistic effect on HCT116 cells [125].

## 3. Conclusions

**Figure 2:**
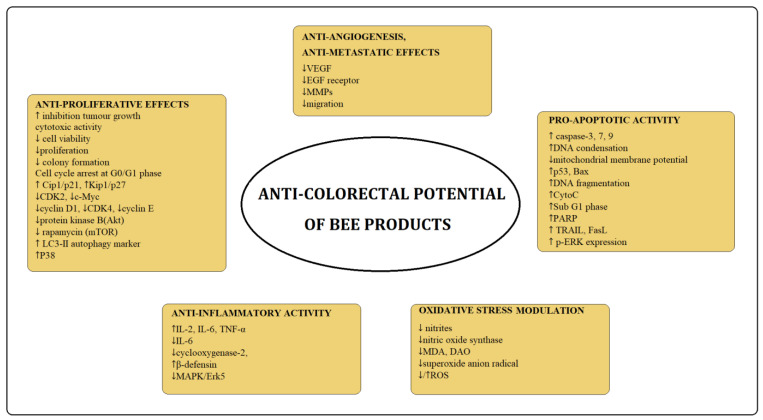
Targets of phytochemicals from bee products on colorectal cancer cells (↑—increase; ↓—decrease).

This review provides insight into the role of bee products in the management of colon cancer. Bee products such as propolis, honey, royal jelly, and bee venom are sources of bioactive components with anti-cancer potential (Figure 2).

Most of the research concerns the activity of honey and propolis; much less is known about the activity of other bee products. It should be noted that the anticancer mechanisms of action of active ingredients present in bee products are not fully understood and require further research and analysis, with particular emphasis on human intervention studies. The obtained research results would provide important insight into the therapeutic use of bee products and would also allow the development of effective actions for the prevention and treatment support of colon cancer. Another problem in the use of bee products in the pharmaceutical industry is the issue of standardization and proper dosage, which should be confirmed in clinical trials.

## Data Availability

Detailed data available from the authors.
